# Specific Protein Quantification by Radioimmuno-Dot-Blot Assay for Complex Mixture Samples Utilizing Strep-Tag and Tritium-Labeled Strep-Tactin

**DOI:** 10.1021/acs.analchem.4c03393

**Published:** 2025-01-07

**Authors:** Maaria Malkamäki, Julie-Anne Gandier, Kristoffer Meinander, Markus B. Linder

**Affiliations:** † Department of Bioproducts and Biosystems, School of Chemical Engineering, Aalto University, 00076 Aalto, Finland; ‡ The Centre of Excellence in Life Inspired Hybrid Materials (LIBER), Aalto University, 00076 Aalto, Finland

## Abstract

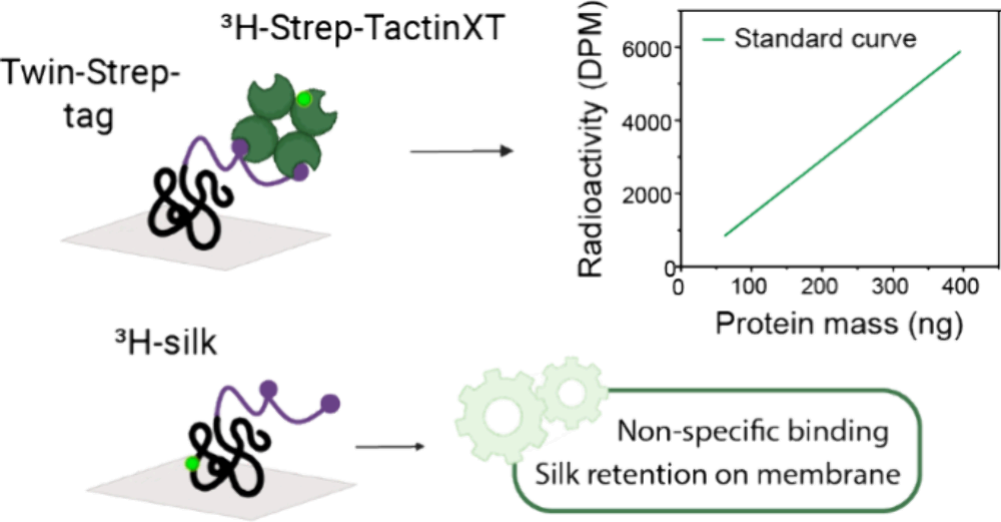

Accurately quantifying specific proteins from complex mixtures like cell lysates, for example, during in vivo studies, is difficult, especially for aggregation-prone proteins. Herein, we describe the development of a specific protein quantification method that combines a solid-state dot blot approach with radiolabel detection via liquid scintillation counting. The specific detection with high sensitivity is achieved by using the Twin-Strep protein affinity tag and tritium-labeled ^3H^Strep-TactinXT probe. While the assay was developed with the recombinant silk protein CBM-AQ12-CBM as a target, the method can be adapted to other recombinant proteins. Variations of the protein tag and Strep-Tactin probe were tested, and it was found that only the combination of Strep-TactinXT and Twin-Strep-tag performed adequately: with this combination, a precision of 95% and an accuracy of 86% were achieved with a linear region from 19 to 400 ng and a limit of quantification at 0.4 pmol. To achieve this, critical optimization steps were preventing nonspecific adsorption and promoting surface adhesion of the target protein to the solid nitrocellulose membrane. The often-overlooked challenges of sample preparation and protein immobilization in quantification assays are discussed and insights into overcoming such issues are provided.

## Introduction

To reliably determine protein concentrations, careful consideration of the conditions under which a particular method is applied is required. Sample preparation steps, suitable sample volume and concentration, detection range, protein chemistry (e.g., amino acid sequence), cosolutes, and buffer composition are all features that may impact the ability to accurately quantify protein concentrations and therefore need to be taken into consideration when choosing the most appropriate method.^1−5^

Spider silk is an exceptional biomaterial with great mechanical properties, biocompatibility, and biodegradability. Recombinant production of silk is one way toward applications, but more understanding of the material formation is needed.^6−9^ Therefore, in our case, we require accurate specific protein quantification from complex mixtures, for example, cell lysate, to support both in vivo and in vitro studies, with as little sample preparation as possible, which excludes the use of many conventional quantification methods.^1−3,10^

Specific solid-state protein quantification methods exist, and the most commonly used are Western blot and preceding radio-immunoblot. However, with a dot-blot approach, the procedure can be simplified compared to Western blot and reproducibility challenges can be minimized.^11−14^ Additionally, by using liquid scintillation counting (LSC) as a detection method, the challenge of signal saturation both in luminescence and fluorescence detection of Western blots and autoradiography of radio-immunoblots can be avoided while preserving good differentiation from the background.^11,15−17^ Advantages of LSC are that the linear region is wide and measurements are fast.^11,12,15,17−22^ The radiolabel for LSC detection can be attached to any protein, widening the study possibilities beyond antibody-dependent systems and also allowing for direct labeling of protein-of-interest (POI).^23−26^

Peptide tags or affinity tags, like Strep tags, can be used in recombinant quantification systems. The strep-tag-streptavidin binding complex originates from the biotin and streptavidin natural high-affinity binding complex, which is one of the strongest natural noncovalent binding complexes.^27^ Engineered versions of both tag and streptavidin have been developed for recombinant protein studies. Strep-tagII is an eight amino acid containing affinity tag, whereas Twin-Strep-tag contains the same eight amino acid sequence twice with an intermediate linker sequence. Strep-Tactin and Strep-TactinXT are engineered versions of natural streptavidin with small mutations to improve specific binding to Strep-tagII. The different tag-probe pairs have been reported to have binding affinities from ∼0.3 μM for Strep-tagII with Strep-Tactin, improving to ∼70 pM for Twin-Strep-tag with Strep-TactinXT.^28^ The development of Twin-Strep-tag has widened the applicability to protein detection and quantification but the reported systems use only streptavidin or Strep-Tactin lacking possible improvements by Strep-TactinXT.^29−32^ The Strep-tag and probe system can be attached to any recombinant protein with the same high specificity, and both tag and probe can be produced in a recombinant manner avoiding possible specificity, cross-reactivity, and production challenges related to native antibody or tag-antibody systems.^23,24,29−31,33−40^ Additionally, the strep system is not restricted to any specific detection method but can be coupled with desired detection methods like liquid scintillation counting via covalently bound radiolabel.^17,25,26,41,42^

Here, we present a new specific protein quantification method that combines a solid-state dot blot approach with sensitive radiolabel detection by liquid scintillation counting. High specificity is achieved with an N-terminal encoded protein Twin-Strep affinity tag and tritium-labeled ^3^H-Strep-TactinXT. The method is developed using recombinant silk protein AQ12 (engineered version of ADF3 of garden spider *Araneus diadematus*) with cellulose binding modules (CBM) as terminal domains as a model protein.^43^ The method can be adapted to other proteins to which a Twin-Strep tag can be fused. Additionally, we provide a more general framework for optimizing dot blot assays and highlight assay steps that can create a significant variation in the results of immunoassay protocols generally.

## Results and Discussion

### Radioimmuno-Dot-Blot Assay Development and Evaluation

**Figure 1:**
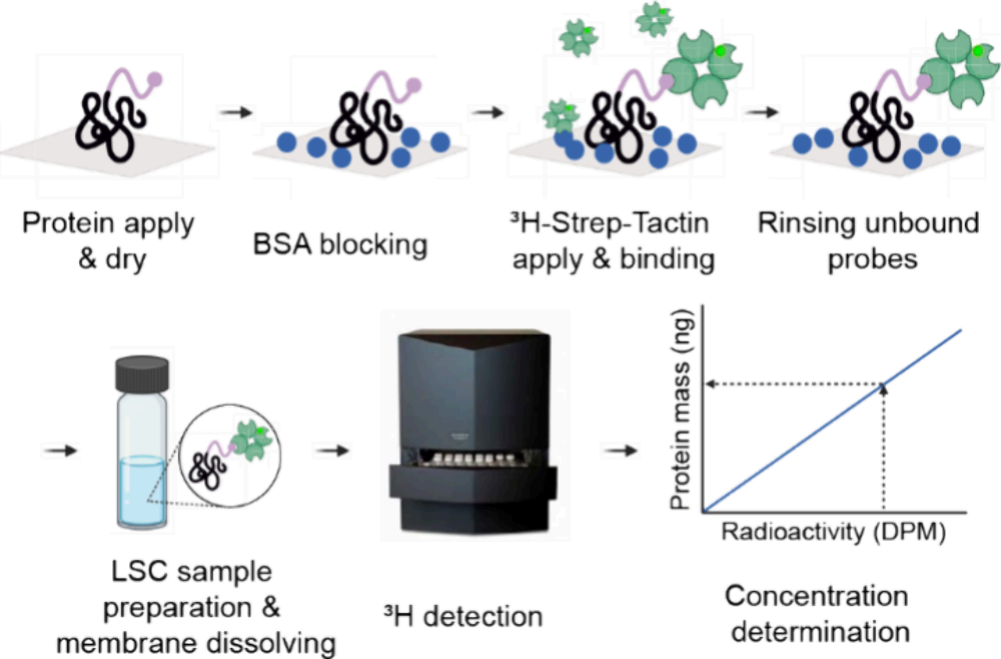
Radio-method workflow following steps of a Western dot-blot: applying a protein solution on the membrane and drying it, blocking the membrane with BSA, probing the POI with ^3^H-Strep-Tactin, rinsing unbound probes, preparing the LSC sample by placing a sample membrane into a scintillation cocktail in which the membrane dissolves, detecting tritium activity with a liquid scintillation counter, and constructing a resulting standard curve that can be used to determine the concentration of unknown samples.

A radioimmuno-dot-blot assay was developed based on the classic workflow: the solution containing the specific protein to be quantified (referred to herein as protein of interest or POI) is pipetted directly onto a membrane which is then dried and treated with a blocking solution—such as bovine serum albumin (BSA)—to prevent or minimize nonspecific binding in further steps. Next, the sample is selectively labeled and quantified with a probe (Figure 1).^44^ Here, the POI was engineered to contain a Twin-Strep-tag, which is detected by its association with a tritium-labeled probe (^3^H-Strep-TactinXT). The tritium bound to the POI was quantified by LSC to obtain the disintegration count per minute (DPM). The amount of POI was determined using a standard curve for DPMs of known amounts of the probe. A detailed protocol is presented in Materials and Methods.

**Figure 2:**
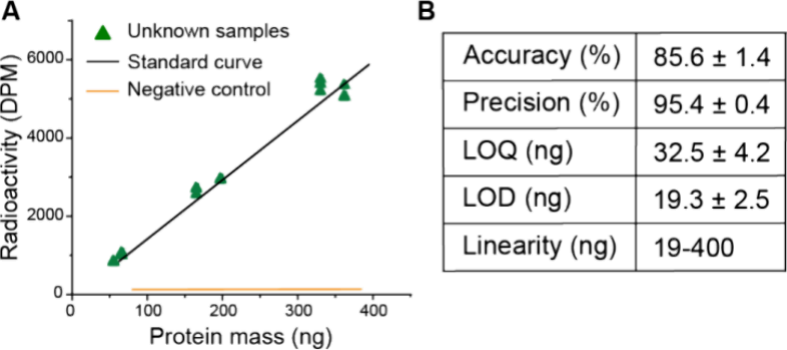
Final method. (A) Standard curve with unknown samples demonstrating the accuracy of quantification. (B) Validation parameters.

The performance of the assay was evaluated with a set of experiments (Figure 2). The precision of quantification was determined to be 95% by using the relative standard deviation of replicate samples. The accuracy was determined by comparing the measured concentration of an unknown sample to a reference sample determined independently using UV–visible spectroscopy and amino acid analysis. The relative error in determining the concentration of unknown samples was 14%, giving an accuracy of 86%. The precision and accuracy are most prone to user error. During sample preparation, the dilution steps must be extremely carefully conducted to minimize errors in accuracy. Likewise, when applying the sample on the membranes, careful and repeated pipetting—such that in each sample and during each assay, it is performed exactly the same way—decreases the relative standard deviation and therefore increases the precision. The limit of detection (LOD) and quantification (LOQ) were determined by using the low-concentration region. The values were determined by comparing the detected values to the background level of the negative control samples. LOD was determined to be 19.3 ng (0.2 pmol). It is the point where the DPM value difference between the actual sample and negative control is three times the standard deviation of negative control samples. For LOQ, a difference of 10 times the standard deviation of the negative control was used, giving 32.5 ng (0.4 pmol) as the value. The linear region was determined with the help of statistical tests, and the range of 19–400 ng was linear with statistical significance. The detection limits and concentration differentiation ability are also highly dependent on the labeling yield of tritium compound with Strep-Tactin. By optimizing the labeling reaction, the quantification limit and concentration differentiation limit could be improved further to the low pM region.^28^

**Figure 3:**
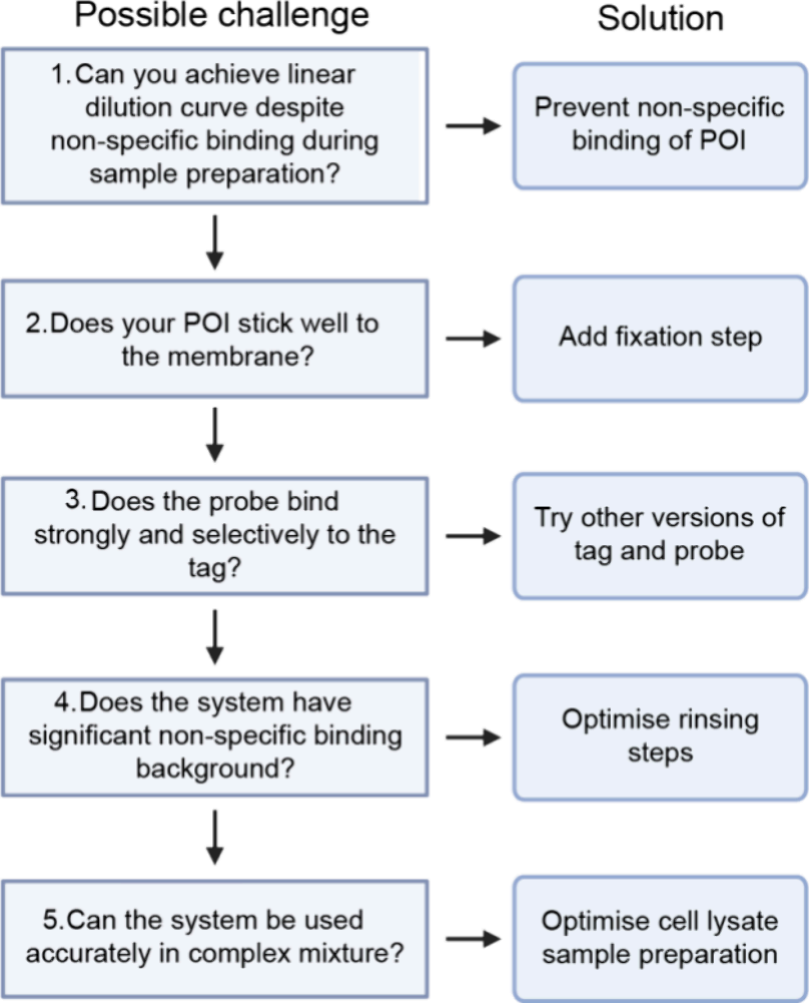
Flowchart of challenges addressed in the optimization process.

Optimization of individual steps was required to achieve a functioning assay. The steps and their possible challenges are listed in Figure 3. To aid optimization, Twin-Strep-CBM-AQ12-CBM was directly labeled with tritium to enable its quantification after each step. This labeled silk (^3^H-silk) enabled us to study the dilution curve and standard curve preparation during sample preparation as well as silk protein retention on nitrocellulose membranes. It also allowed for studying nonspecific binding on sample membranes. In addition, we studied tag and probe binding, as well as detection from complex mixtures. The optimization of these steps (Figure 3) is presented in the subsequent sections. The studied silk proteins were our POI, Twin-Strep-CBM-AQ12-CBM, as normal and labeled version (^3^H-silk) and CBM-AQ12-CBM (a protein without the Twin-Strep-tag) as negative control.

### Nonspecific Binding in Sample Preparation

**Figure 4:**
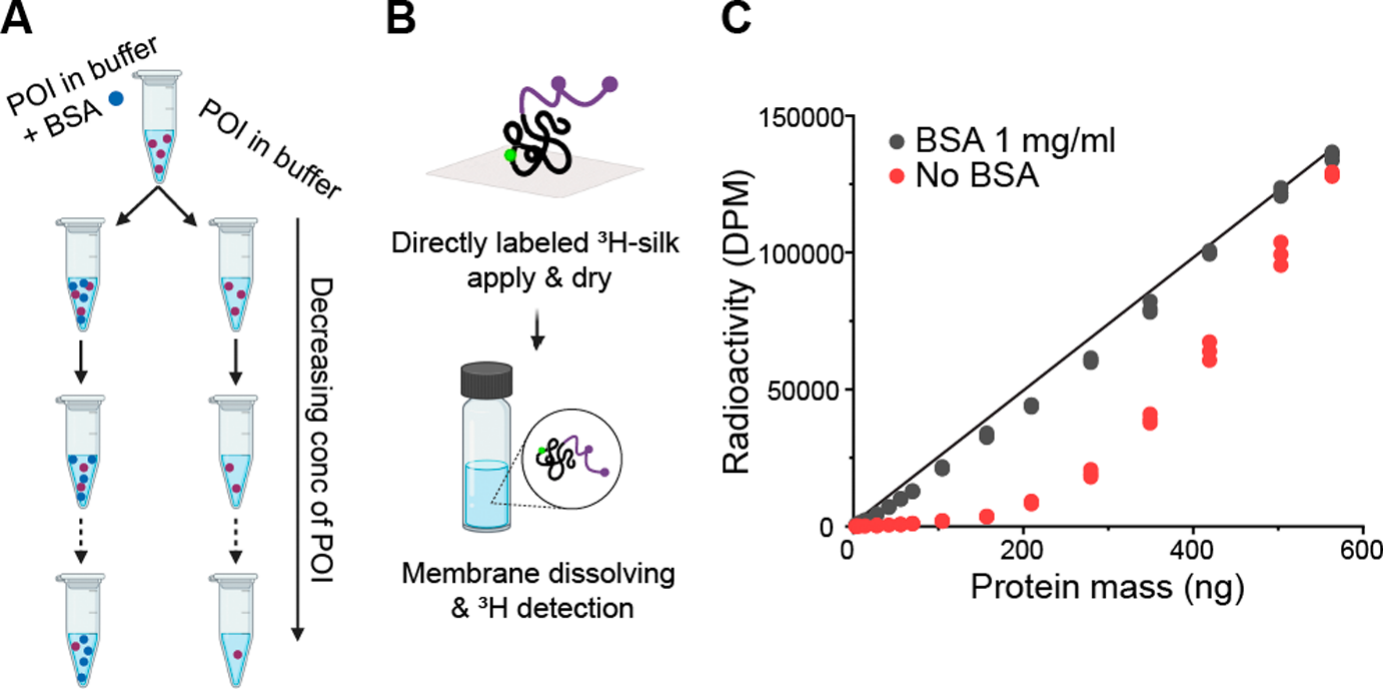
Nonspecific binding in sample preparation. (A) Schematic of the sample preparation of a dilution series with BSA in addition to POI and a series with only POI. (B) Schematic of the assay part of an experiment; ^3^H-silk was applied onto a membrane, which was measured directly. (C) Graph showing the nonspecific binding during sample preparation without the addition of BSA, which results in a loss of the POI during dilutions.

To evaluate the extent of nonspecific binding of proteins to sample tubes and pipet tips during sample preparation, a dilution series of ^3^H-silk was prepared. The solutions were applied to a nitrocellulose membrane, and the radioactivity was detected using a scintillation cocktail that dissolved the membrane (Figure 4A,B). We see a significantly bent dilution curve, where silk protein was lost during the dilution steps due to nonspecific binding (Figure 4C, red data points). To prevent nonspecific binding, we performed the dilution steps using a buffer which contained 1 mg/mL BSA. This allowed for the preparation of linear dilution curves for standards and reduced loss in preparing samples for analysis. The linear dilution curve is presented in Figure 4C (black data points). Different proteins do have varying tendencies for nonspecific binding, but the possibility should be considered generally for proteins at low concentrations in assays.

### Protein Retention on the Membrane

**Figure 5:**
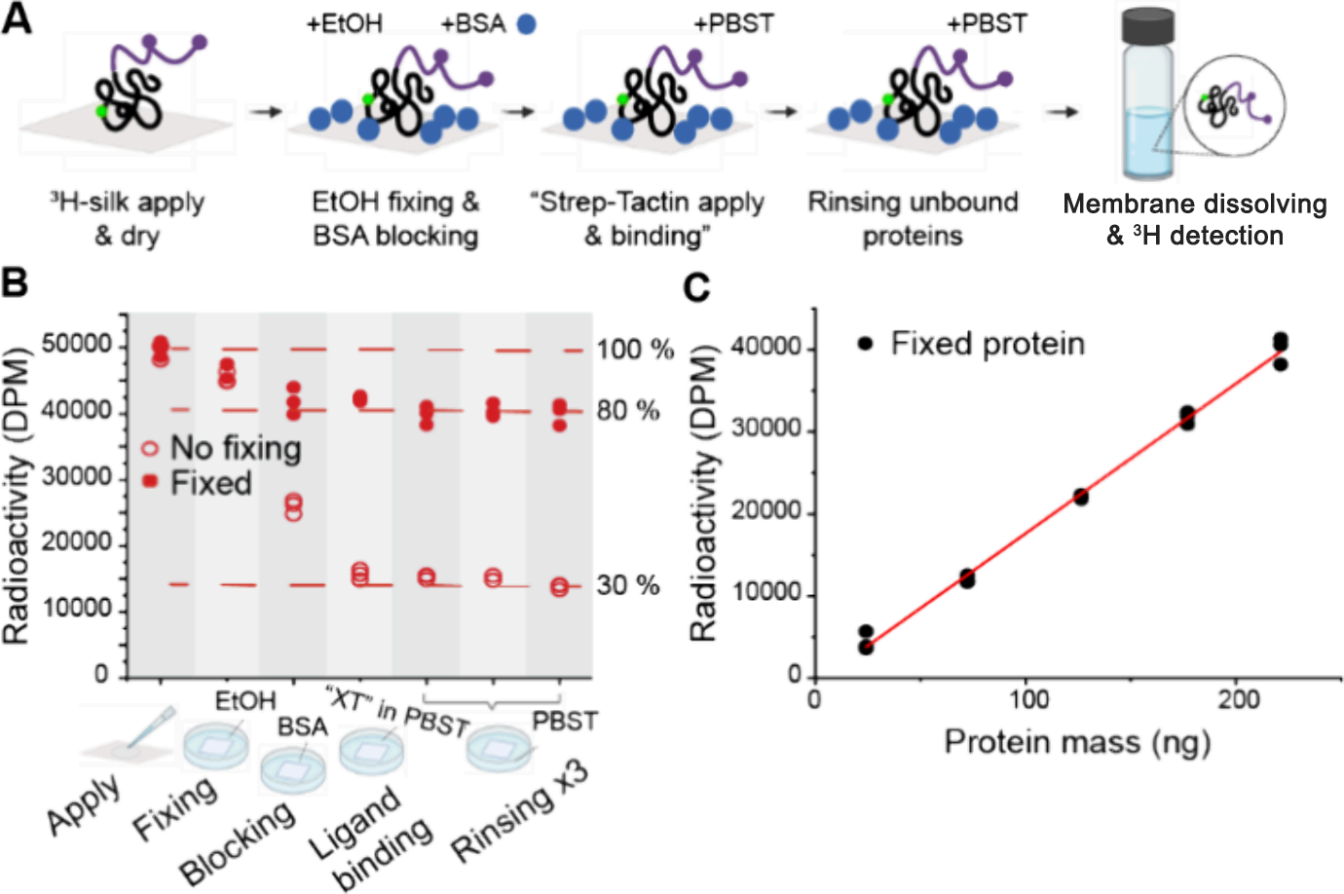
Binding and retention of silk with fixation versus with no fixation. (A) Workflow of the experiment with ^3^H-silk. (B) Protein loss during assay steps with ethanol fixation versus no fixation. (C) Retention of silk after fixation is in percentage constant over concentrations, and a linear standard curve can be prepared.

To determine the binding strength of the POI to the nitrocellulose membrane, its retention was studied using ^3^H-silk. Samples for quantification were taken after each assay step starting from the initial protein application and throughout the rinsing steps (Figure 5A). The fixing step was not part of the original protocol but was added based on retention test results. When fixation was tested, controls (nonfixed samples) were incubated in water instead of ethanol. During the Strep-Tactin binding step, all samples were incubated in PBST.

The results show (Figure 5B) a significant loss of ^3^H-silk without fixing (open circles). Especially during blocking and probe binding steps, a large amount of ^3^H-silk protein desorbs from the membrane. The desorption continues even during the final rinsing steps, but because blocking and probe binding steps have the longest duration, the effect is more pronounced there. After the whole assay, only 30% of the original applied ^3^H-silk protein was left on the membrane. It has been reported that hydrophobic interactions are crucial in protein–membrane binding, indicating worse binding of hydrophilic proteins, like silk proteins, to nitrocellulose membrane,^45−49^ but the extent of loss was not expected, and this has not been widely discussed. This large desorption could significantly affect the reproducibility and linearity of the assay and reduce the detection limit. Other POIs might bind naturally stronger to the membrane; however, it is necessary to consider desorption for all POIs even if they are expected to bind to nitrocellulose well. To improve POI retention on the membrane, a fixation step can be added, and ethanol proved to be an effective fixation agent for silk protein. Also, glutaraldehyde and methanol were tested as fixation agents. Glutaraldehyde fixed our POI effectively but reacted with our Strep-tag, preventing the detection interaction of the tag and Strep-Tactin. Methanol did not show advantages over ethanol. With ethanol fixation, the retention of ^3^H-silk was increased to approximately 80% of the original applied amount (Figure 5B filled circles), and a proportionally similar retention was obtained in different POI concentrations resulting in a linear curve after all assay steps (Figure 5C). The optimal fixation method is likely to be highly dependent on the nature of the POI which should be kept in mind while optimizing the retention. It is also important to keep in mind that the retention will be highly unlikely to ever be 100%, even with extensive optimization of fixation.

**Figure 6:**
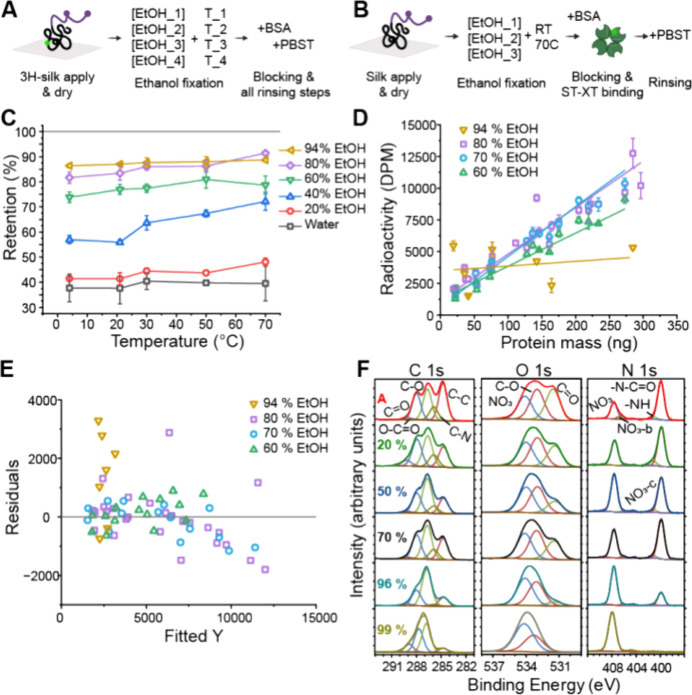
Fixing optimization. (A) Schematic presentation of optimization of the retention experiment with ^3^H-silk. (B) Schematic of optimization of the tag functionality-experiment with normal silk and labeled probe. (C) Optimization of ethanol fixation to achieve the best possible retention of silk protein using varying fixation conditions. (D) Optimization of ethanol fixation to achieve the best possible tag functionality in combination with high retention by varying ethanol fixation conditions. (E) Residual plot from linear fits in plot D highlighting differences between different ethanol solutions. (F) XPS spectra from surface of fixed silk on nitrocellulose membrane explaining the effect of high concentration ethanol on tag functionality. The differently colored rows represent samples fixed with different ethanol concentrations (%) and A as a silk reference with no treatments.

Ethanol fixation still required optimization, as we found that different ethanol concentrations and temperatures influenced the yield. First, only the retention of ^3^H-silk to the membrane was optimized by trying various ethanol concentrations at different temperatures from +4 to 70 °C. Following the parallel fixation at different conditions, the remaining steps from blocking to final rinsing were carried out identically (Figure 6A). Strep-Tactin was not used and was replaced by PBST-buffer. The retention yield was particularly affected by the ethanol concentration and by the temperature (Figure 6C). A higher retention yield for ^3^H-silk is achieved with an increasing ethanol concentration. A major increase in retention is obtained already from 20% ethanol to 40% and continuing further to 60%, but after that, the retention still improves with higher ethanol concentration. In the best case, over 90% retention is achieved. Overall, higher temperature seems to give slightly better retention.

In addition to retention yield of ^3^H-silk, retaining also the tag functionality for probe binding was still verified. Here, higher ethanol concentrations (60, 70, 80, and 94%) were tested at two temperatures: 21 °C (i.e., room temperature, RT) and 70 °C. Normal silk was used with labeled Strep-TactinXT for probe binding with all other steps included (Figure 6B). Surprisingly, the temperature seemed to have an adverse effect, and at 70 °C, the results were worse than in RT. Therefore, room temperature was chosen for the final fixation temperature.

Unexpectedly, the tag functionality was strongly affected by the fixation conditions. In Figure 6D, the tag functionality results are shown for fixation at room temperature for different ethanol concentrations. While the highest ethanol concentration (94%) was the best option for maximizing retention of ^3^H-silk as measured directly, for tag functionality, it performed the worst. The activity values are overall random and widely scattered as can be seen from the large residual values (Figure 6D,E). A high ethanol concentration therefore prevents the tag-probe binding from functioning properly. However, at 60–80% ethanol concentration, the tag probe binding is functional. Between 70 and 80% ethanol fixation, there is no significant difference in the tag functionality performance, but 70% treated samples have a slightly greater slope, and the linearity is also better based on smaller residuals compared to 80% ethanol-treated samples. 70% ethanol at room temperature was therefore chosen as the final fixation method. Without the direct detection of ^3^H-silk, this desorption loss would have been difficult to identify, especially since protein retention and tag availability responded so differently. To understand the surprising loss of tag-probe binding at a high concentration of ethanol, the surface of the silk samples on the nitrocellulose membrane was studied with X-ray photoelectron spectroscopy (XPS). In Figure 6F, Supplementary Figure S1, and Supplementary Tables S1–S5, the gradual decrease of C–C and N–C=O peaks in the C 1s and N 1s spectra, respectively, as well as an increase of the NO_3_ peak in the N 1s spectra with increased ethanol concentration indicate a decrease of silk and an increase of nitrocellulose on the outermost layer of the sample surface. As the ^3^H detection showed that the protein was still present in the membrane, we find it likely that a higher ethanol concentration in fixation causes a migration of the silk deeper into the nitrocellulose matrix and therefore limits the tag availability. The change in peak heights and O/C ratio (Table S1) between 70 and 94% ethanol-treated samples is the largest concluding the significant difference in tag functionality between 70 and 94% samples. Additionally, the ethanol treatment might change the secondary structure of silk.^50−53^

### Tag and Probe Selection

**Figure 7:**
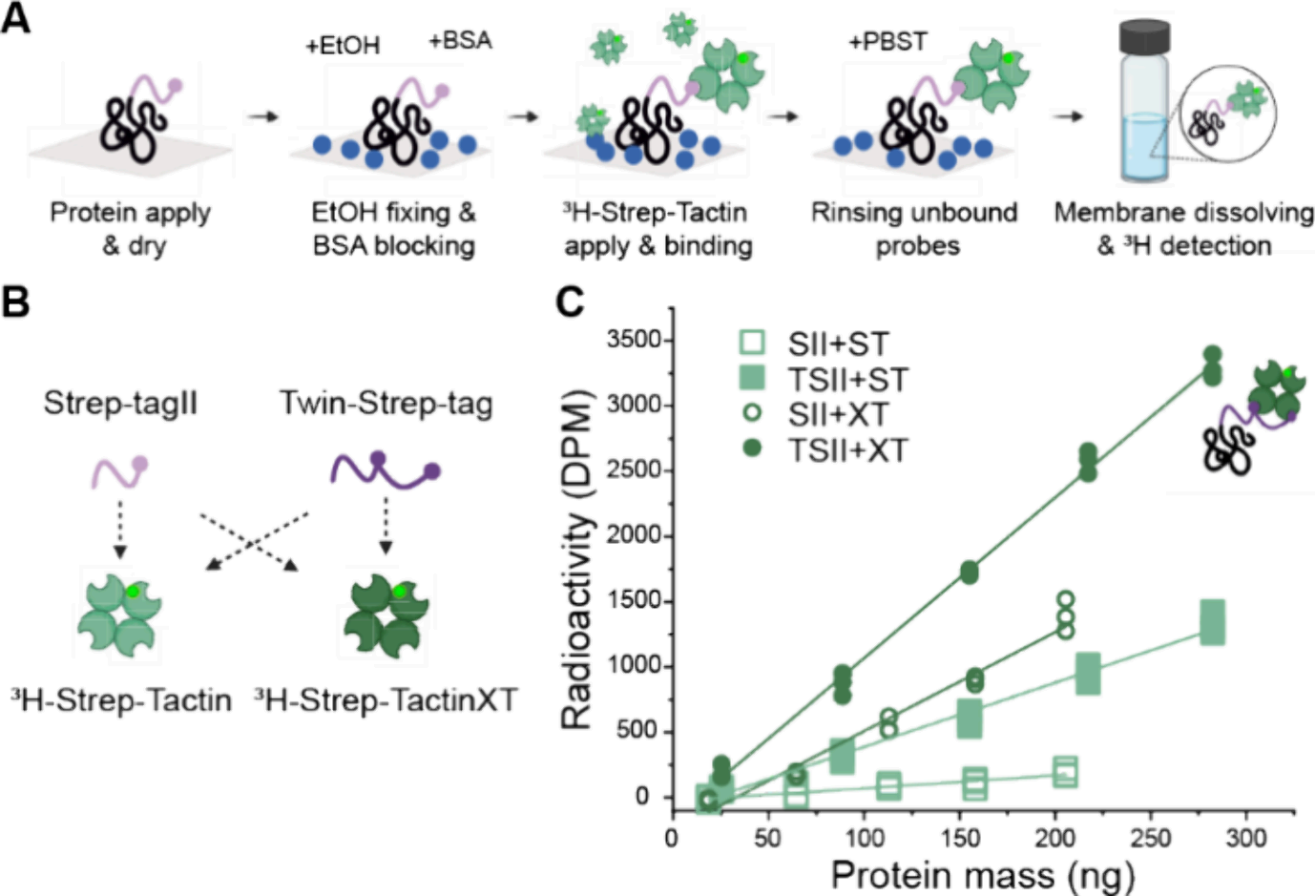
Tag and probe combination comparison. (A) Schematic of experiment assay with normal silk and labeled Strep-Tactin. (B) Illustration of different tags and probes that were compared in the experiment. (C) Graph presenting Twin-tag (filled symbols) with Strep-TactinXT (circles) as the best combination for the assay.

Different engineered versions of the Strep-tag and Strep-Tactin were tested to achieve the best possible specificity and concentration differentiation. The whole assay was conducted for a dilution series of silks with different Strep-tag in the N-terminal using also different Strep-Tactin versions in the probe binding step (Figure 7A). The different tested tag versions were Strep-tagII and Twin-Strep-tag, which contain the Strep-tagII sequence twice with a linker in between. From the probe part, Strep-Tactin and Strep-TactinXT were tested. Illustrations of the different tags and probes and the used combinations are presented in Figure 7B. The effect of the varied combinations can be seen in Figure 7C. Strep-TactinXT gives a greater slope compared to Strep-Tactin regardless of tag. For the same probe, the Twin-Strep-tag also has a greater slope than the Strep-tagII. Thus, the combination of Twin-Strep-tag and Strep-TactinXT gives the best results and was chosen for the final assay. This combination has the best binding stability and sensitivity due to the highest activity values, greatest slope, and linear response. The specificity is also best due to a low background from the negative control (data not shown here). This confirms that the Twin-Strep tag with Strep-TactinXT is a sufficient combination for a quantification assay. The other combinations were clearly inferior choices for analytical purposes, but they may be useful in other applications as, for example, purification tags.^28^

In addition to the tag and probe-pair selection, the concentration of the probe has a strong effect on the dynamic range of detection and needs to be optimized. Four probe concentrations were tested with molar ratios of 0.75, 1.12, 1.50, and 3.00 of the probe to Twin-Strep-tag calculated for 30 ng/μL silk concentration (Table 1). For the dilution series of silk protein, the same probe concentration for all silk concentrations was used, meaning that the molar ratio of the probe to tag depends on the silk concentration. Low probe concentrations give a low background and do not restrict detection at the lower end. Therefore, better quantification is achieved at the low end but the linear range is limited at the higher end. Higher probe concentration increases the slope and, therefore, improves concentration differentiation. Also, the linear range expands at the high end. With high probe concentration, on the other hand, the background activity starts to increase, and therefore the lower-end detection limits increase, leading to a decrease in sensitivity. The probe concentration has also minor effects on the *R*^2^ value of the standard line and on the precision via relative standard deviation, with the best values achieved with intermediate probe concentrations. Therefore, a compromise based on one’s needs must be made. It is possible to optimize a few different concentration quantification regions for different purposes with different probe concentrations. In our case, a 1.25 molar ratio of the probe-to-tag was chosen as the final concentration. This concentration gives a higher slope than the lower concentrations, theoretical limit of detection (based on linear equation and background level) very close to 0 ng, linearity up to ∼400 ng of silk, a very small standard deviation in percentage, and still a low background.

**Table 1:** Probe Concentration Optimization

molar ratio (probe/tag)	linearity high end (ng)	standard and neg. control intersection (ng)	slope	*R*^2^	SD%
3.00	>350	9	63.6	0.977	4.92
1.50	>350	–1	50.7	0.9838	3.66
1.12	300	–1	45.3	0.9793	3.13
0.75	300	–20	35.1	0.9665	3.81

Even with a highly specific probe, nonspecific binding must be minimized and optimized with rinsing steps. Three rounds of rinsing were found to be sufficient (Supplementary Figure S3). Nonspecific binding was also studied in detail by analyzing the location of binding on the sample (Supplementary Figure S4). Nonspecific binding occurs mostly at the edge of the sample membrane where silk is not present.

### Complex Mixture Samples

**Figure 8:**
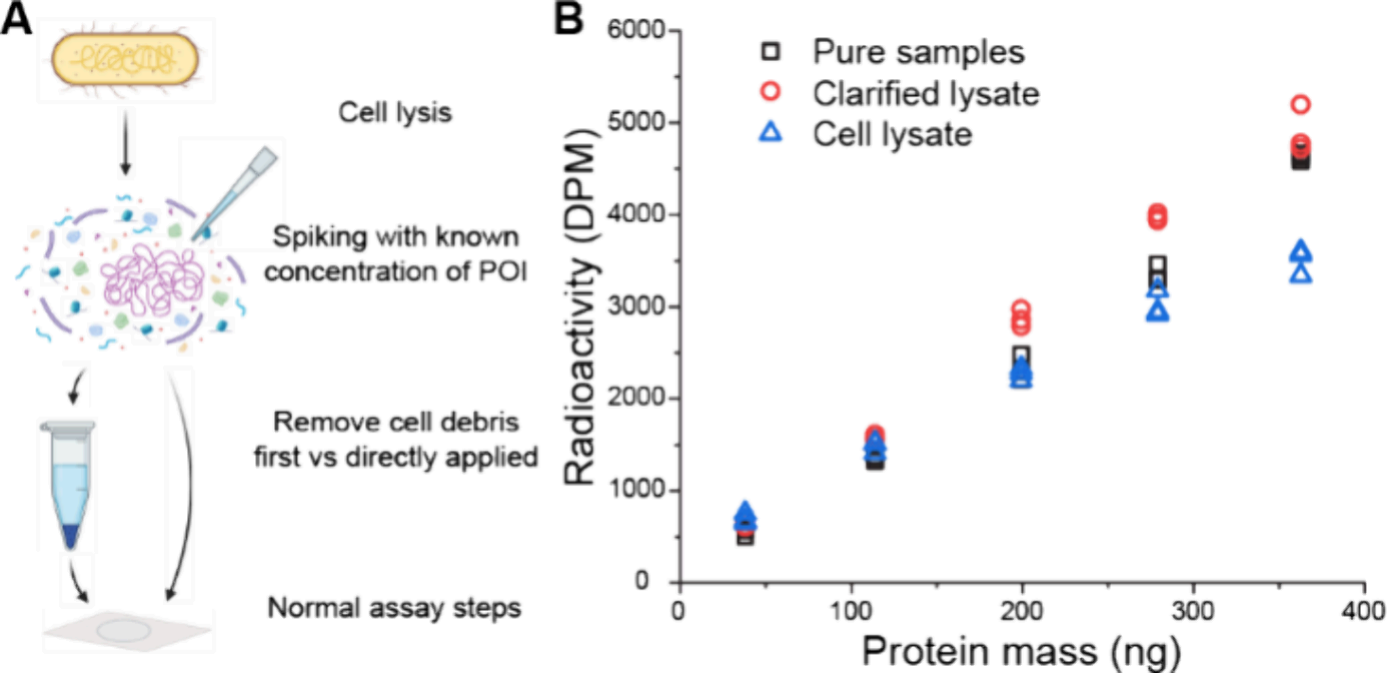
Optimization of cell lysate experiments. (A) Schematic of the experiment. (B) Graph presenting the comparison of crude cell lysate, clarified lysate, and pure protein samples resulting in the need for removal of cell debris from the samples. Without clarification, the exposure of tag is limited at high concentrations, as seen in the cell lysate-series.

The ability to quantify the POI from complex mixtures was tested with cell lysate samples. *E. coli* cells were lysed and spiked with known concentrations of Twin-Strep-silk, and the activities were compared to a parallel series of purified standard Twin-Strep-silk (Figure 8A). The effect of cell debris from cell lysis was also tested by comparing the activities to clarified cell lysate. After initial sample preparation, the assay was conducted as normal. In Figure 8B, we see that the cell debris of the cell lysate has a clear effect on the linear range of the quantification. The curve for cell lysate samples starts to bend at higher concentration samples. This is likely due to the lysed bacteria pieces being so large that they cover the sample and limit the exposure of POI. Only at the lowest concentration point is the cell lysate so diluted that it does not affect the quantification. Therefore, the clarification of the lysate is needed for accurate quantification. After clarification, the linearity of the curve is restored, and the activity overlaps with the pure (standard) samples. The presence of other soluble proteins and DNA in the clarified lysate slightly improves the binding of Strep-TactinXT at higher concentrations, and the specificity of Strep-TactinXT-tag binding is not affected by cellular proteins. This possibility of accurate quantification from a complex mixture in the solid state is a clear advantage of the method and allows in vivo studies of proteins and other protein mixture studies.

### Other Important Aspects

In addition to the optimization of components and concentrations for the assay, seemingly small details can significantly affect the accuracy and repeatability of the quantification assay. During almost all of the assay steps, the sample membrane is treated with some sort of solution. For fixation, blocking, probe binding, and rinsing, it is crucial that the POI and membrane are in good contact and properly immersed in these solutions. If the assay is conducted in, for example, 1.5 mL tubes, the membrane is locked into one position, and the solutions effectively affect only the inner surface of the membrane. In tubes, the membrane is also in a vertical orientation, which might affect the binding and rinsing yields. We found that an assay conducted on well plates gives higher activity values with still a small percentage standard deviation, as the membrane is fully immersed and can freely move during rocking.

The membrane orientation plays a role, even if the membrane can move freely in the solution. If the membrane is placed upside down on the well so that the POI side faces the bottom, the activity values at the end are lower. This indicates that fixation or probe binding does not work efficiently on the bottom side of the well. This could be improved by increasing the volume of solutions used in the assay, but careful control of placing the membranes POI-side up prevents this and simultaneously reduces reagent consumption.

After optimization of the most accurate binding of POI and probe, high accuracy of the quantification must also be ensured by the correct choice of the liquid scintillation cocktail. Generally, scintillation cocktails are good but are developed for specific usage. In this assay, nitrocellulose membranes are used, and dissolving them is preferred for accurate detection. Only some cocktails are designed for dissolving nitrocellulose, and the Filter Count by PerkinElmer worked well here. Dissolving is not immediate and requires a few hours with initial sample inverting to ensure the submergence and start of dissolution.

When applying this method for quantification, it is recommended to use the same protein or at least as similar as possible for the standard curve than for what is the protein of interest. If the standard protein and measured proteins are significantly different, the binding geometry of probe to tag might change in addition to possible retention yield changes, and thus, it needs to be considered. In addition, the nonspecific binding during sample preparation and protein retention on the sample membrane is recommended to be assessed when using different proteins. Those steps are recommended to be assessed also in all quantification assays to ensure accurate and reproducible results. Ideally, with all quantification assay optimizations, one should have the pure protein and label it to verify all the steps of the assay, focusing especially on the immobilization and nonspecific binding steps.

### Proof of Concept

**Figure 9:**
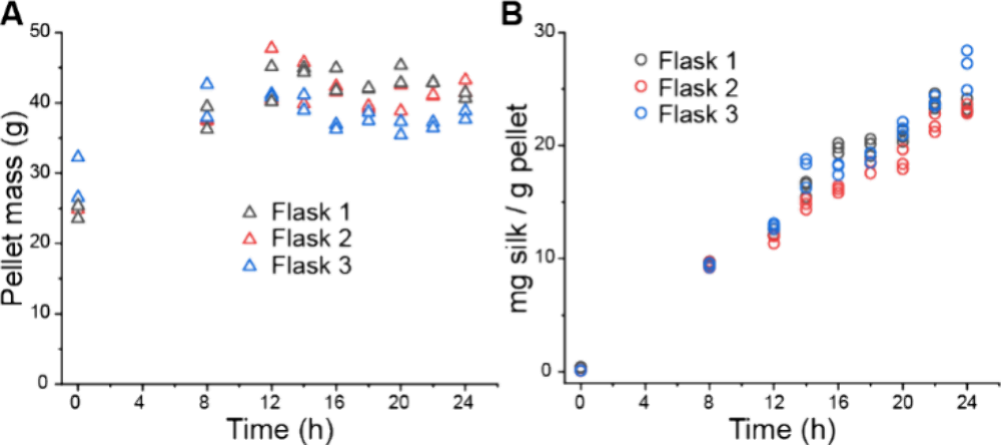
Proof of concept: protein production assay. (A) Cell growth curve during protein production (g cells/L of production media). (B) Protein production curve from induction to harvest (mg protein produced/g cells in pellet).

As a proof of concept, a production curve of Twin-Strep-CBM-AQ12-CBM in *E. coli* was constructed. We determined concentrations of POI at different time points, starting at induction with IPTG and continuing for 24 h of production and until final purification. The last 12 h of the production are the most interesting; thus, the sampling rate was increased from 12 to 24 h. In Figure 9A, the cell mass per ml of media, and in Figure 9B, the silk protein yield per gram of cells in the pellet over time is presented. The cell mass increases for the first 12 h after the induction, but after that, it stabilizes and slightly decreases during the last 12 h of production. The protein production starts at the induction and the protein yield per mass of cells increases nearly linearly until the end point at 24 h. The replicate flasks behave similarly, having only slight variation in the yield toward the end of the production. The small variation especially between flasks 2 and 3 highlights how the higher recombinant protein overproduction limits other cell functionalities as cell growth in flask 3 and results in lower cell mass whereas with slightly lower protein production yield the cell mass can reach slightly higher yield.^54^ The results show that POI production can continue well after the cell mass increase has ended.

After purification by affinity chromatography, samples were prepared and quantified by amino acid analysis (AAA) and compared to the results of the quantification assay from the last time point samples. These results are presented in Table 2. The results of AAA are approximately one-third of the result of quantification assay, which highlights the loss of protein during the purification process. When comparing the samples from different production flasks, the order in magnitude is the same between AAA and the quantification assay, validating the accuracy of the results.

**Table 2:** Purified Samples Analyzed with Amino Acid Analyzer (AAA) and Compared to the Quantification Assay at the End Point of Production as mg of Protein per mL of Production Media

sample	AAA (mg/mL)	quantification assay (mg/mL)
1	0.302	0.973
2	0.297	0.962
3	0.336	1.012

## Conclusions

We developed a specific protein quantification dot-blot method for complex mixtures with solid-state detection utilizing Twin-Strep-tag and Strep-TactinXT specific tag-probe binding and sensitive radioactivity detection by liquid scintillation counting. The method was developed using recombinant silk protein but is possible to adapt to other recombinant proteins that can be modified with a Twin-Strep-tag. This new method benefits studies with aggregation-prone proteins such as silk proteins and is a good tool for in vivo studies of recombinant protein systems and other complex mixtures. Our optimization process was greatly strengthened by using directly labeled POI enabling the study of individual assay steps separately. A comparable optimization would be very difficult to implement with other detection methods. The directly labeled POI highlighted the importance of preparative first steps of the assay in controlling error sources. It revealed that nonspecific binding during sample preparation (i.e., dilution series) and poor retention of silk protein on nitrocellulose are significant factors in assay performance and are factors to be considered in any other protein assay as well. By using BSA in sample preparation, the accuracy of quantification in lower concentrations can be restored, and with an additional fixation step, the retention can be significantly improved. Unexpectedly, there was a compromise between immobilization efficiency and tag availability. Additionally, the Twin-Strep-tag in combination with Strep-TactinXT was found to be the only sufficient tag-probe pair for protein quantification. Although the needed equipment for the method may not be available in every laboratory, the method ideas and optimization steps provide ideas and support for all assay users and improve the quality of other optimized assays.

## Materials and Methods

### Materials

The buffer compositions, protein constructs, and protein sequences are provided in the Supporting Information.

### Methods and Instruments

The experimental methods and instrumentation details for protein expression, total amino acid composition analysis (AAA), ultraviolet–visible spectroscopy, tritium labeling of silk and probe, and X-ray photoelectron spectroscopy (XPS) are provided in the Supporting Information.

### Radioimmuno-Dot-Blot Assay Protocol from Complex Mixture Samples

The protocol contains the use of radioactive tritium and may require special permission for radioactive work. Immediately before conducting the assay, the samples of known concentration will serve to establish the standard curve. BSA is used to prevent nonspecific binding. 10 μL aliquot of clarified cell lysate (or pure protein in the case of the standard) by micropipette was placed onto a nitrocellulose membrane (1.35 cm × 1.35 cm, pore size 0.45 μm) and then left to dry under ambient conditions. Once dry, the sample membranes were transferred to 12-well plates (well bottom area = 3.85 cm^2^, diameter ∼2.2 cm, nontreated) and the protein was fixed to the membrane by incubating with 500 μL of 70% (v/v) ethanol at room temperature for 10 min. To block nonspecific binding sites on the nitrocellulose membrane, incubation (stationary) was done with 500 μL of blocking buffer at room temperature for 1 h. The sample membranes were incubated for 1 h in PBST containing ^3^H-Strep-TactinXT at the appropriate molar ratio for the anticipated concentration range (see Table 1). Incubation was done on a gently rocking plate. The membranes (stationary) were washed three times for 5 min (total of 15 min) with PBST. Each sample membrane was dissolved in 4 mL of scintillation cocktail (Filter Count) in a 5 mL sample tube followed by inverting the tube head over tail 10 times to ensure the membranes were entirely submerged, wetted with the liquid, and dissolved effectively. To ensure an accurate measurement, complete dissolution of the membranes is required (approximately 6 h). They are then mixed (inverting head over tail 10 times) before being placed on the scintillation counter. Tritium activity in the samples was detected and quantified by measuring disintegrations per minute (dpm) with the appropriate measurement settings for the scintillation counter being used.

### Liquid Scintillation Counting

To quantify labeled proteins, we used liquid scintillation counting (Hidex 300 SL). A Filter Count scintillation cocktail (PerkinElmer) was used for sample preparation in all cases. This scintillation cocktail can dissolve the nitrocellulose membrane, which is essential for reliable results of the samples on the membrane. 4 mL of the scintillation cocktail with the sample membrane was added to 5 mL plastic vials (Hidex), and tubes were inverted 10 times to ensure complete submergence and start of dissolution. Membranes were allowed to dissolve from 6 h to 18 h to ensure full dissolution and were inverted again prior to measurement. Measurement blanks were prepared without any radioactive compound and measured as the first and last samples to ensure proper function of the machine. Each sample was measured for 300 s, and the TDCR values were monitored to ensure data quality. Measured DPM values were used for result analysis and converted to concentrations with the help of a standard curve.

### Limit of Detection and Limit of Quantification

To validate the detection and quantification limit, a signal-to-noise ratio method was used.^55^ A dilution series for standard protein Twin-Strep-CBM-AQ12-CBM was prepared with a range of 0.99–55 ng, and negative control samples were prepared at the same concentration range from CBM-AQ12-CBM. The standard deviation of background samples was determined and multiplied by 3 to obtain the limit of detection and by 10 to obtain the limit of quantification value. The obtained value was added to the background activity value, and based on this number, a mass was determined based on the linear equation of the standard curve. The equations are presented in Supporting Information eqs 1 and 2.

### Linearity and Linear Region

To determine the linearity of the standard curve and the linear range of detection, both visual and statistical analysis was performed. Visual observation was done for data ranging from 1 to 1000 ng and statistical analysis for a range of 40–500 ng. Linear regression curves were determined for data series based on the sum of least-squares, and the linearity was evaluated with the Analysis of Variance lack-of-fit (LOF) test and comparing the resulting *F*-value to the tabulated value. The null hypothesis (*H*_0_) of the test is that there is no lack-of-fit (regression is linear), whereas the alternative hypothesis (*H*_A_) states that lack-of-fit is present, and some nonlinear regression should be applied. Therefore, when the calculated *F*-value is smaller than the tabulated *F*-value, the null hypothesis is true, and a linear equation best describes the data.^55,56^ The equations used for the calculations are presented in Supporting Information eqs 3–12.

### Accuracy and Precision

The reference method used to determine the accuracy of the dot blot method was UV–visible spectroscopy. Accuracy of the assay was expressed with help of the relative error.^55^ Replicate samples were used to determine the precision and were expressed with the help of the relative standard deviation of replicate samples. Accuracy and precision were determined across the linear region of the response curve. The equations used for the calculations are presented in Supporting Information eqs 13–18.

## Data Availability

All newly generated data are presented in the manuscript and the Supporting Information. The raw data are available at Zenodo.org: DOI: 10.5281/zenodo.12579698.

## Supporting Information Available

Buffer compositions; protein constructs; experimental procedures for protein expression and purification, total amino acid composition analysis (AAA), ultraviolet–visible spectroscopy, tritium labeling of silk, tritium labeling of probe, and X-ray photoelectron spectroscopy (XPS); protein sequences of Twin-Strep-CBM-AQ12-CBM, StrepII-CBM-AQ12-CBM, and CBM-AQ12-CBM; optimization of nonspecific binding of probe; location of nonspecific binding on sample membranes; and calculations of validation parameters (PDF)
