# Supplementary material for: Specific Protein Quantification by Radioimmuno-Dot-Blot Assay for Complex Mixture Samples Utilizing Strep-Tag and Tritium-Labeled Strep-Tactin
Source: Anal Chem. 2025 Jan 7;97(2):1087–96. doi: 10.1021/acs.analchem.4c03393 (PMC11755393; doi:10.1021/acs.analchem.4c03393)
Supplement: Supplementary file 1 — ac4c03393_si_001.pdf [file ac4c03393_si_001.pdf]

Supporting Information

Specific protein quantification by radioimmuno-dot-blot assay for complex mixture samples utilizing Strep-tag and tritium-labelled Strep-Tactin

Maaria Malkamäki[a,b], Julie-Anne Gandier[a,b], Kristoffer Meinander[a,b], and Markus B. Linder[a,b]*

[a]Department of Bioproducts and Biosystems, School of Chemical Engineering, Aalto University, FI-00076 Aalto, Finland;

[b]The Centre of Excellence in Life Inspired Hybrid Materials (LIBER), Aalto University, 00076 Aalto, Finland

* Corresponding author:

Email: markus.linder@aalto.fi

Table of Contents

Buffer compositions S3

Protein constructs S3

Protein expression in *E. coli* and purification S3

Total amino acid composition analysis (AAA) S4

Ultraviolet-visible spectroscopy S4

Tritium labelling of Twin-Strep-CBM-AQ12-CBM S4

Tritium labelling of Strep-Tactin and Strep-TactinXT S4

X-ray photoelectron spectroscopy (XPS) S5

Twin-Strep-CBM-AQ12-CBM sequence S5

StrepII-CBM-AQ12-CBM sequence S5

CBM-AQ12-CBM sequence S6

X-ray photoelectron spectroscopy of sample membranes S6

Non-specific binding of probe S10

Location of non-specific binding on sample membranes S12

Calculations of validation parameters S13

References S15

Buffer compositions

Phosphate-buffered saline with Tween (PBST): KH_2PO_4 4mM (Sigma), Na_2HPO_4 16mM (Sigma), NaCl 115mM (VWR Chemicals), Tween20 0.05% (Sigma), pH 7.4

Blocking buffer: 3 % Bovine serum albumin (BSA, Sigma, >98%) in PBST

Tris-HCl: 50 mM Tris-HCl (Trizma base, Sigma), 100mM NaCl (VRW Chemicals), pH 7.4

Lysis buffer: 50 ml Tris-HCl-buffer, 75 μl 2M MgCl2 (Sigma), protease inhibitor tablet (Pierce protease inhibitor tablet, EDTA-free, Thermo Scientific), lysozyme (Thermo Scientific), DNaseI (Sigma)

Protein constructs

Three different silk-inspired proteins were studied, each differing only in the amino acid sequence of the N-terminal tag. The silk-inspired protein sequence was designed with a triblock structure: a highly repetitive sequence in the middle and globular terminal domains at both N and C termini. The repetitive mid-block was an engineered version of the major ampulla gland silk fibroin 3 (ADF3) from *A. diadematus* called AQ12 that contains 12 repeats derived from ADF3. [1] The globular terminal domains were cellulose-binding modules CBM3 from *Ruminiclostridium thermocellum*. [2] His6-tag was part of the protein at the C terminus. The three different variants had at the N terminus either no tag, Strep-tagII, or Twin-Strep-tag (Figure S1). The cloning procedure of the no-tag and Strep-tagII protein has been described earlier. [3,4] The Twin-Strep-tag was synthesized by GenScript into pUC57-Mini plasmid and attached to the N terminus of no tag proteins sequence using restriction enzymes XbaI and NcoI (Thermo Fischer Scinetific). The protein sequences can be found in their own sections later in the Supporting information.

Two different probe proteins were used for the specific detection: Strep-Tactin and its engineered version Strep-TactinXT (IBA Lifesciences), and they were ordered in 5 mg quantities.

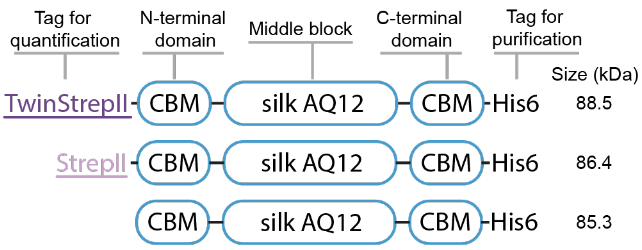

Figure S1. Schematic structures of the silk protein structures used in this study.

Protein expression in *E. coli* and purification

For protein expression, an EnPresso B500 medium (EnPresso) was used. After cell lysis, preliminary purification of the recombinantly expressed protein was carried out by heating the crude extract to 70 C for 30 min followed by centrifugation to remove the precipitated impurities. More precise purification was carried out with immobilized metal affinity chromatography (IMAC, ÄKTA pure by GE Healthcare) using nickel as the metal (nickel affinity chromatography) and proteins C-terminal His6-tag as binding agent. The protein was eluted with an imidazole-containing buffer (Binding buffer: 500mM NaCl, 20mM Imidazole, pH 7.4, Elution buffer: 500mM NaCl, 500mM Imidazole, pH 7.4) and gel-filtrated into deionized water using EconoPac 10 DG columns (Bio-Rad).

Total amino acid composition analysis (AAA)

To determine the exact protein concentration of stock proteins used for the standard curves, amino acid analysis was performed. The ÄKTA purified protein sample was hydrolyzed in 6 M HCl (Merck), containing 0.1% phenol (Sigma-Aldrich) at 110 C for 24 h. L-Norleucine (Sigma-Aldrich) was used as an internal standard. After the hydrolysis, the acid was evaporated. The remaining sample was resuspended according to the system protocol (Sykam GmbH) and filtered, and the supernatant was analyzed with an amino acid analyzer (S433, Sykam GmbH) with a UV detector at 570 nm and 440 nm. Protein amounts were calculated based on the internal standard and the protein sequence. Glycine, alanine, glutamine, and glutamic acid were used for the concentration determination since they are most abundant in the protein and therefore most reliable.

Ultraviolet-visible spectroscopy

To determine the exact protein concentration of thawed stock proteins for individual experiments, ultraviolet-visible spectroscopy was performed. The stock proteins were diluted to 0.1 mg/ml protein concentration based on the amino acid analyzed stock concentration and the absorbance was measured with a quartz cuvette with a path length of 1 cm. The molar extinction coefficients for the proteins were obtained from the Expasy ProtParam tool based on the protein sequences.

Tritium labelling of Twin-Strep-CBM-AQ12-CBM

Tritium labelling of Twin-Strep-CBM-AQ12-CBM for studying the early steps of the assay was carried out with [^{3}H]Nsuccinimidylpropionate ([^{3}H]-NSP) (Novandi). The labelling reaction must be conducted in special laboratory space with permission for radioactive work. Further, 5 mCi (185 MBq) of [^{3}H]-NSP stored in a heptane:ethyl acetate solution (3:2) was used. The solvent was evaporated close to dryness using a nitrogen stream. The highly concentrated solution was mixed with 6.5 ml of a protein solution in Sodium bicarbonate buffer ($NaHCO_3$ 100 mM, pH 8.3). The concentration of the protein solution was 3.4 gL^{-1}. The reaction was conducted at room temperature for 3.5 h. The solution was gently mixed at reaction time points 0.5, 1, 1.5, and 2.5 h. The unreacted label was removed, and the labelled protein was buffer exchanged to phosphate buffered saline (PBS: KH_2PO_4 4mM, Na_2HPO_4 16mM, NaCl 115mM, pH 7.4) with Econo-Pac 10 DG desalting columns (Bio-Rad). The final activity of labelled protein was determined by a liquid scintillation counter. The final protein concentration was determined by ultraviolet-visible spectroscopy.

Tritium labelling of Strep-Tactin and Strep-TactinXT

Tritium labelling of Strep-Tactin and Strep-TactinXT for quantification of the silk proteins with the assay was carried out with [^{3}H]N-succinimidylpropionate ([^{3}H]-NSP) (Novandi). The labelling reaction must be conducted in special laboratory space with permission for radioactive work. Further, 5 mCi (185 MBq) of [^{3}H]-NSP stored in a heptane:ethyl acetate solution (3:2) was used for Strep-Tactin and 1 mCi for Strep-TactinXT. Most of the solvent was evaporated using a nitrogen stream. The concentrated solution was mixed with 4 ml of Strep-Tactin and 1.5 ml of Strep-TactinXT in Sodium bicarbonate buffer ($NaHCO_3$ 100 mM, pH 8.3). The concentration of Strep-Tactin was 1.25 gL^{-1} and the

concentration of Strep-TactinXT was 0.67 gL^{-1}. The solution was mixed at room temperature for 4 h. The unreacted label was removed, and the labelled protein was buffer exchanged to phosphate buffered saline (PBS: KH_2PO_4 4mM, Na_2HPO_4 16mM, NaCl 115mM, pH 7.4) with Econo-Pac 10 DG desalting columns (Bio-Rad), and the final activity was determined by a liquid scintillation counter. The final protein concentration was determined by ultraviolet-visible spectroscopy.

X-ray photoelectron spectroscopy (XPS)

To study the effect of high-concentration ethanol treatment on the silk samples and tag functionality, X-ray photoelectron spectroscopy was conducted. The measurements were performed with a Kratos AXIS Ultra DLD X-ray photoelectron spectrometer using a monochromatic $Al_{K\alpha}$ X-ray source (1486.7 eV) run at 100 W. A pass energy of 80 eV and a step size of 1.0 eV were used for survey spectra, while high-resolution spectra were acquired with a pass energy of 20 eV and a step size of 0.1 eV. Photoelectrons were collected at a 90° take-off angle under ultra-high vacuum conditions, with the base pressure of the system typically below 1×10^{-9} Torr. The X-ray beam spot had a diameter of 1 mm, while the area of analysis was 300 × 700 µm. Photoemission spectra were collected from three different spots on each sample surface, from which average values were calculated for all results presented here. A relative error of less than 3 % was found for the average concentrations of the most abundant elements, indicating that the analyzed areas of the samples were very homogeneous. All acquired spectra were charge-corrected relative to the position of C–C bonding at 284.8 eV.

Twin-Strep-CBM-AQ12-CBM sequence

Structure: Twin-Strep-tag – Terminal domain (CBM) – linker - Middle part (AQ12) - linker – Terminal domain (CBM) – His-tag

MSAWSHPQFEKGGGSGGGSGGSAWSHPQFEKAMGNLKVEFYNSNPSDTTNSINPQFKVTNTGSSAIDLSKLTLR
YYYTVDGQKDQTFWCDHAAIIGSNGSYNGITSNVKGTFVKMSSSTNNADTYLEISFTGGTLEPGAHVQIQGRFAKN
DWSNYTQSNDYSFKSASQFVEWDQVTAYLNGVLVWGKEPSASASASAGASAAASAGAGAGAGPYGPGASAAA
AAAGGYGPGSGQQGPGQQGPGQQGPGQQGPGQQGPYGPGASAAAAAAGGYGPGSGQQGPGQQGPGQQG
PGQQGPGQQGPYGPGASAAAAAAGGYGPGSGQQGPGQQGPGQQGPGQQGPGQQGPYGPGASAAAAAAG
GYGPGSGQQGPGQQGPGQQGPGQQGPGQQGPYGPGASAAAAAAGGYGPGSGQQGPGQQGPGQQGPGQ
QGPGQQGPYGPGASAAAAAAGGYGPGSGQQGPGQQGPGQQGPGQQGPGQQGPYGPGASAAAAAAGGYGP
GSGQQGPGQQGPGQQGPGQQGPGQQGPYGPGASAAAAAAGGYGPGSGQQGPGQQGPGQQGPGQQGPG
QQGPYGPGASAAAAAAGGYGPGSGQQGPGQQGPGQQGPGQQGPGQQGPYGPGASAAAAAAGGYGPGSGQ
QGPGQQGPGQQGPGQQGPGQQGPYGPGASAAAAAAGGYGPGSGQQGPGQQGPGQQGPGQQGPGQQGP
YGPGASAAAAAAGGYGPGSGQQGPGQQGPGQQGPGQQGPGQQASASASAAASAASTVANSSSNLKVEFYNS
NPSDTTNSINPQFKVTNTGSSAIDLSKLTLRYYYTVDGQKDQTFWCDHAAIIGSNGSYNGITSNVKGTFVKMSSSTN
NADTYLEISFTGGTLEPGAHVQIQGRFAKNDWSNYTQSNDYSFKSASQFVEWDQVTAYLNGVLVWGKELEHHHH
HH

StrepII-CBM-AQ12-CBM sequence

Structure: Strep-tagII – Terminal domain (CBM) – linker – Middle part (AQ12) – linker – Terminal domain (CBM) – His-tag

MWSHPQFEKGMGNLKVEFYNSNPSDTTNSINPQFKVTNTGSSAIDLSKLTLRYYYTVDGQKDQTFWCDHAAIIGS
NGSYNGITSNVKGTFVKMSSSTNNADTYLEISFTGGTLEPGAHVQIQGRFAKNDWSNYTQSNDYSFKSASQFVEW
DQVTAYLNGVLVWGKEPSASASASAGASAAASAGAGAGAGPYGPGASAAAAAAGGYGPGSGQQGPGQQGPG
QQGPGQQGPGQQGPYGPGASAAAAAAGGYGPGSGQQGPGQQGPGQQGPGQQGPGQQGPYGPGASAAAA
AAGGYGPGSGQQGPGQQGPGQQGPGQQGPGQQGPYGPGASAAAAAAGGYGPGSGQQGPGQQGPGQQGP
GQQGPGQQGPYGPGASAAAAAAGGYGPGSGQQGPGQQGPGQQGPGQQGPGQQGPYGPGASAAAAAAGG
YGPGSGQQGPGQQGPGQQGPGQQGPGQQGPYGPGASAAAAAAGGYGPGSGQQGPGQQGPGQQGPGQQ
GPGQQGPYGPGASAAAAAAGGYGPGSGQQGPGQQGPGQQGPGQQGPGQQGPYGPGASAAAAAAGGYGPG
SGQQGPGQQGPGQQGPGQQGPGQQGPYGPGASAAAAAAGGYGPGSGQQGPGQQGPGQQGPGQQGPGQ
QGPYGPGASAAAAAAGGYGPGSGQQGPGQQGPGQQGPGQQGPGQQGPYGPGASAAAAAAGGYGPGSGQQ
GPGQQGPGQQGPGQQGPGQQASASASAAASAASTVANSSSNLKVEFYNSNPSDTTNSINPQFKVTNTGSSAIDL
SKLTLRYYYTVDGQKDQTFWCDHAAIIGSNGSYNGITSNVKGTFVKMSSSTNNADTYLEISFTGGTLEPGAHVQIQ
GRFAKNDWSNYTQSNDYSFKSASQFVEWDQVTAYLNGVLVWGKELEHHHHHH

CBM-AQ12-CBM sequence

Structure: Terminal domain (CBM) – linker – Middle part (AQ12) – linker – Terminal domain (CBM) – His-tag

MGNLKVEFYNSNPSDTTNSINPQFKVTNTGSSAIDLSKLTLRYYYTVDGQKDQTFWCDHAAIIGSNGSYNGITSNV
KGTFVKMSSSTNNADTYLEISFTGGTLEPGAHVQIQGRFAKNDWSNYTQSNDYSFKSASQFVEWDQVTAYLNGV
LVWGKEPSASASASAGASAAASAGAGAGAGPYGPGASAAAAAAGGYGPGSGQQGPGQQGPGQQGPGQQGP
GQQGPYGPGASAAAAAAGGYGPGSGQQGPGQQGPGQQGPGQQGPGQQGPYGPGASAAAAAAGGYGPGSG
QQGPGQQGPGQQGPGQQGPGQQGPYGPGASAAAAAAGGYGPGSGQQGPGQQGPGQQGPGQQGPGQQG
PYGPGASAAAAAAGGYGPGSGQQGPGQQGPGQQGPGQQGPGQQGPYGPGASAAAAAAGGYGPGSGQQGP
GQQGPGQQGPGQQGPGQQGPYGPGASAAAAAAGGYGPGSGQQGPGQQGPGQQGPGQQGPGQQGPYGP
GASAAAAAAGGYGPGSGQQGPGQQGPGQQGPGQQGPGQQGPYGPGASAAAAAAGGYGPGSGQQGPGQQ
GPGQQGPGQQGPGQQGPYGPGASAAAAAAGGYGPGSGQQGPGQQGPGQQGPGQQGPGQQGPYGPGASA
AAAAAGGYGPGSGQQGPGQQGPGQQGPGQQGPGQQGPYGPGASAAAAAAGGYGPGSGQQGPGQQGPGQ
QGPGQQGPGQQASASASAAASAASTVANSSSNLKVEFYNSNPSDTTNSINPQFKVTNTGSSAIDLSKLTLRYYYTV
DGQKDQTFWCDHAAIIGSNGSYNGITSNVKGTFVKMSSSTNNADTYLEISFTGGTLEPGAHVQIQGRFAKNDWS
NYTQSNDYSFKSASQFVEWDQVTAYLNGVLVWGKELEHHHHHH

X-ray photoelectron spectroscopy of sample membranes

The decrease in tag functionality after the highest ethanol concentrations could originate from changes in silk protein orientation on the nitrocellulose membrane in a way that the tag is not available for probe binding. This would be visible as a changed surface layer of the sample which can be analyzed with X-ray photoelectron spectroscopy (XPS). XPS is a very surface-sensitive technique with a maximum probing depth of around 5-10 nm, and an exponential decrease in photoelectron intensity with increasing sample depth. The results show clear differences in the molecular composition of the surface of the sample membranes, especially between samples fixed with 70 % ethanol and 96 % ethanol. These changes indicate a decrease of signal from silk and an increase of signal from nitrocellulose membrane while ethanol concentration increases. Further details of the results are discussed below.

The relative composition of the samples can be seen in Table S1. Gradual change and differences were detected for the samples with increasing fixation ethanol concentration. The values in Table S1-S5 are averages from measurements on three different positions close to the center of the sample on each measured sample. The survey spectra are shown in Figure S2. The sample names indicate the ethanol concentration (%) used for the fixation, with A as an exception referring to a sample with applied and dried silk with no further treatments. As expected, carbon, oxygen, and nitrogen were the main elements of all samples, with trace levels of sodium, sulfur, and chlorine also detected. The main differences between the samples can be noticed from the changes in the ratio between the amounts of oxygen and carbon in the samples, i.e., the O/C ratio, in the second-to-last column of Table S1, with increasing values for the most part related to a larger fraction of the measured intensities coming from nitrocellulose instead of silk.

Table S1. Relative concentrations of elements in all samples. The two final columns give the O/C and N/C ratio for all films.

Sample	C 1s %	Cl 2p %	N 1s %	Na 1s %	O 1s %	S 2p %	O/C	N/C
A	55,18	0,07	12,48	1,40	30,24	0,63	0,55	0,23
20	54,61	0,00	13,11	0,18	31,90	0,19	0,58	0,24
50	51,81	0,00	12,47	0,16	35,33	0,24	0,68	0,24
70	52,33	0,00	13,07	0,19	34,14	0,27	0,65	0,25
96	48,19	0,00	10,51	0,14	41,08	0,07	0,85	0,22
99	41,61	0,00	10,38	0,18	47,83	0,00	1,15	0,25

Table S2 shows the relative ratios of the different components of carbon in samples. The C 1s spectra were fitted with five Gaussian components according to standard tabulated chemical shifts, with peak positions at 284.8 eV (C-C), 285.6 eV (C-N), 286.7 eV (C-O), 287.8 eV (C=O), and 288.9 eV (O-C=O).[5] A gradual decrease of the C-C component can be seen together with an increase in C-O bonding for samples further down in Table S2. A slightly exaggerated amount of carboxyls for the last sample is most likely an effect of a slight surface charging on the sample, causing a shift of peaks toward higher binding energies. Especially, the significant decrease of the C-C component is an indication of a decreased signal from silk due to a transfer of silk deeper into the membrane matrix. The initial decrease of C-C between samples A and 20 can be an indication of a change in the silk orientation but this cannot be concluded when the silk-membrane ratio is changing so significantly.

Table S2. Relative amounts of the different components of carbon, as compared to the total amount of carbon in the samples.

Sample	C (C-C) %	C (C-N) %	C (C-O) %	C (C=O) %	C (O-C=O) %
A	34,11	12,38	27,93	23,75	1,83
20	22,37	12,83	32,57	27,64	4,59

50	20,39	9,10	40,75	26,66	3,10
70	22,73	11,45	37,09	26,27	2,46
96	14,29	2,84	54,86	24,77	3,25
99	6,85	0,00	44,47	36,23	12,44

Three components of nitrogen related to silk protein could be observed in the N 1s region, located at approximately 399.9 eV, 401.3 eV, and 402.3 eV. Most likely these correspond to nitrogen in amides (-N-C=O), amines (-NH), and hydroxylamines (-NOH), respectively[6] In addition to these, the spectra also contained higher binding energy components related to nitrocellulose, with the major component found at an energy of 407.7 eV (NO_3), typical for nitrogen in nitrate, and two minor components at 406.6 eV ($NO_{3\text{-}b}$) and 404.7 eV ($NO_{3\text{-}c}$), with energies that are too high for typical organic bonding, but slightly lower than what can normally be expected for nitrate bonding, possibly the results of other interactions with the nitrate groups. Table S3 gives the relative amounts of each component as compared to the total amount of nitrogen in the samples. Here, the decrease in nitrogen in amides (-N-C=O) and increase in nitrogen in nitrate (NO_3) while increasing fixation ethanol concentration are clear indications of a decrease of silk signal and an increase of nitrocellulose signal.

Table S3. Relative amounts of the different components of nitrogen, as compared to the total amount of nitrogen in the samples.

Sample	N (-N-C=O) %	N (-NH) %	N (-NOH) %	N (NO_3) %	N ($NO_{3\text{-}b}$) %	N ($NO_{3\text{-}c}$) %
A	64,11	3,14	0,00	27,49	4,83	0,43
20	59,34	5,02	0,63	33,32	0,49	1,20
50	50,38	3,78	0,44	41,64	1,31	2,46
70	53,31	3,31	0,31	39,52	1,83	1,72
96	28,30	2,76	0,22	63,24	2,38	3,11
99	0,42	1,16	0,63	92,76	0,71	4,32

The total amount of nitrogen on the sample surfaces, as compared to all elements in the samples, is shown in the first column of Table S4. This amount is split up to differentiate between the parts that are related to nitrogen from nitrate (i.e., from nitrocellulose), and those related to other organic nitrogen, most likely to be solely present in the silk protein on the samples. The final columns give the relative percentages of these two parts. Taken together with the O/C ratio of the samples (typically an O/C ratio of about 0.3 should be expected for silk, while, e.g., pure mononitrocellulose should have a ratio of about 1.17), the variations in the different nitrogen components point towards an increased signal from nitrocellulose and a decrease in the amount of silk protein in the surface layers of the sample for samples further down in Table S4. This may be a surface effect, as photoelectrons from silk protein in deeper layers of the sample will not be detected.

Table S4. Relative concentration of nitrogen, as well as the concentrations of nitrogen in nitrate and other organic nitrogen in the samples. The final two columns give the ratios in atomic percentage for nitrate and other nitrogen.

Sample	N 1s %	Nitrate %-all	N other %-all	Nitrate %	N other %
A	12,48	4,09	8,39	32,75	67,25
20	13,11	4,59	8,52	35,01	64,99
50	12,47	5,66	6,81	45,41	54,59
70	13,07	5,63	7,44	43,07	56,93
96	10,51	7,22	3,29	68,73	31,27
99	10,38	10,15	0,23	97,79	2,21

Table S5 shows the relative amounts of oxygen in the different samples. The O 1s spectra were fitted with three Gaussian components at approximately 531.5 eV, 532.7 eV, and 534.1 eV, these can be attributed to oxygen and carbon double bonds and single bonds, as well as oxygen in nitrate, respectively. The trends in Table S5 tell a similar story to what can be understood from the nitrogen spectra, although smaller differences in energy between the components can result in a larger error.

Table S5. Relative amounts of the different components of oxygen, as compared to the total amount of oxygen in the samples.

Sample	O (C=O) %	O (C-O) %	O (NO3) %
A	37,00	35,41	27,60
20	29,45	38,52	32,03
50	21,48	42,59	35,93
70	24,69	40,17	35,14
96	8,95	46,32	44,73
99	0,00	46,78	53,22

High-resolution scans from the sulfur S 2p region exhibits a single doublet with the $2p_{3/2}$ peak located at approximately 167.7 eV, typical for organic sulfur, while the Na 1s showed a single peak at approximately 1071.5 eV, typical for ionic sodium. All samples contained only very small amounts of these elements, and only these single components were present.

Additionally, high-resolution spectra from the Cl 2p region exhibited a doublet with the $2p_{3/2}$ peak located at approximately 198.0 eV, which is typical for chloride. Chlorine was found in the first sample, and all chlorine was found to be in the same form.[6]

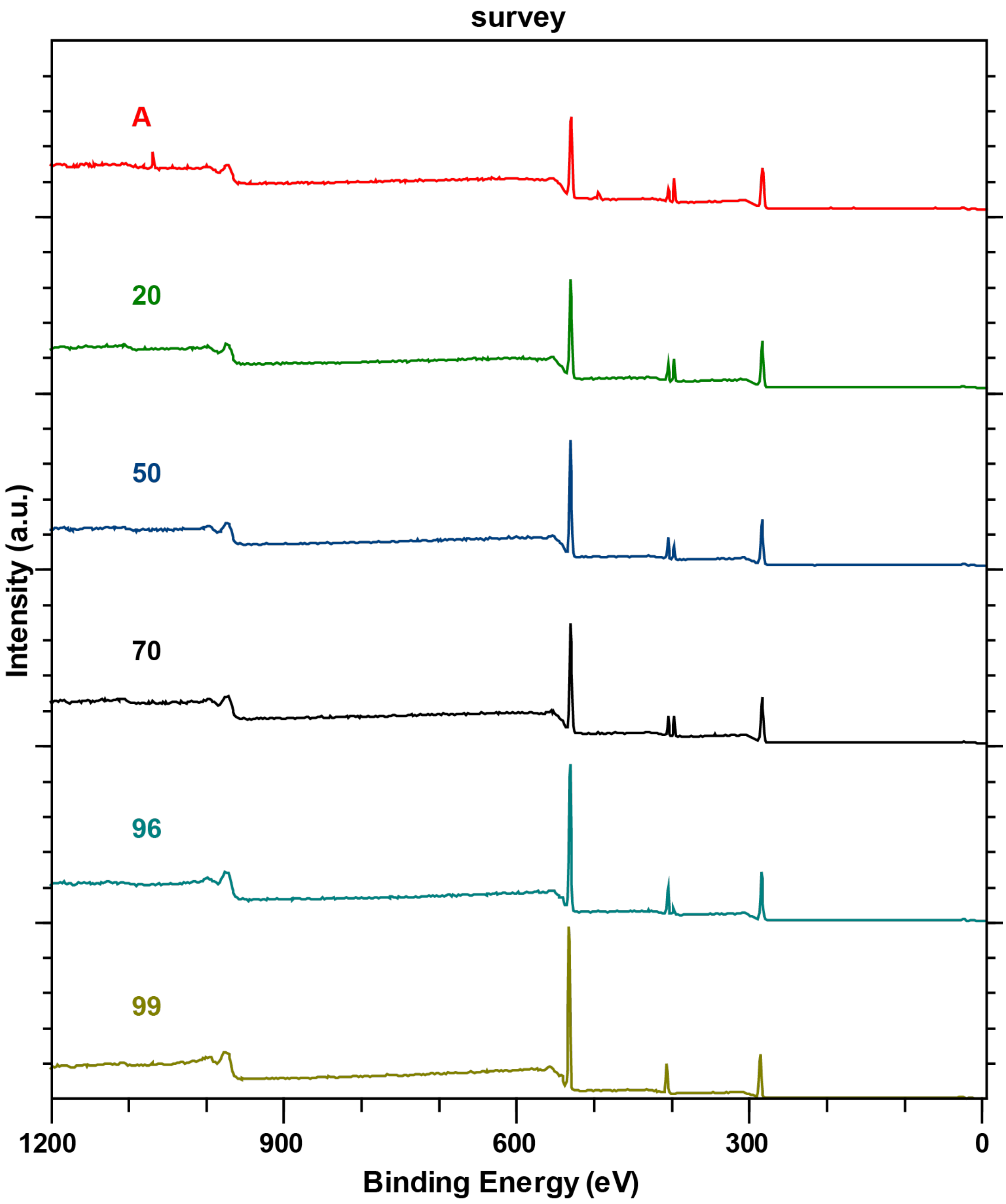

Figure S2. XPS survey spectra of silk sample membranes fixed with different ethanol concentrations (%). A as an exception refers to only applied and dried silk with no further treatments.

Non-specific binding of probe

The required number of rinsing rounds to remove non-specifically bound Strep-TactinXT from the samples was studied by following the activity change over an excess number of rinsing rounds (6x). Rinsing removes the non-specifically bound Strep-TactinXT efficiently, but too extensive rinsing might also remove the Strep-TactinXT probe or silk proteins. Here, the non-specific binding of Strep-TactinXT was studied by varying the rinsing rounds from zero to six (Figure S3A). Non-specific binding was studied with both silks, Twin-Strep-tag, and negative control silk without any tag. In parallel also ^{3}H-silk was used to follow silk retention over rinsing steps (Figure S3B). For ^{3}H-silk, no Strep-TactinXT was added, but otherwise, all the steps were carried out identically.

In Figure S3C, the activities of samples are presented after each rinsing step starting directly after the probe binding step, i.e., 0 rinses. The data series are from high and low concentrations of Twin-Strep-silk and similarly high and low concentrations of negative control (no tag) silk. Rinsing is efficient, and non-specifically bound Strep-TactinXT is removed. After the third rinsing round the activities stabilize, and thus only specifically bound Strep-TactinXT is left in the samples. Three rounds of rinsing are therefore enough to remove the non-specifically bound Strep-TactinXT.

In the negative control, the activities also stabilize after three rounds of rinsing (Figure S3C), leaving a trace of Strep-TactinXT. Importantly the background level is constant. In Figure 8D, the activities from ^{3}H-silk are presented. The activities are stable over all the steps concluding that the silk retention during rinsing steps is constant and the activity decrease in Figure S3C is due to Strep-TactinXT—and not silk—removal.

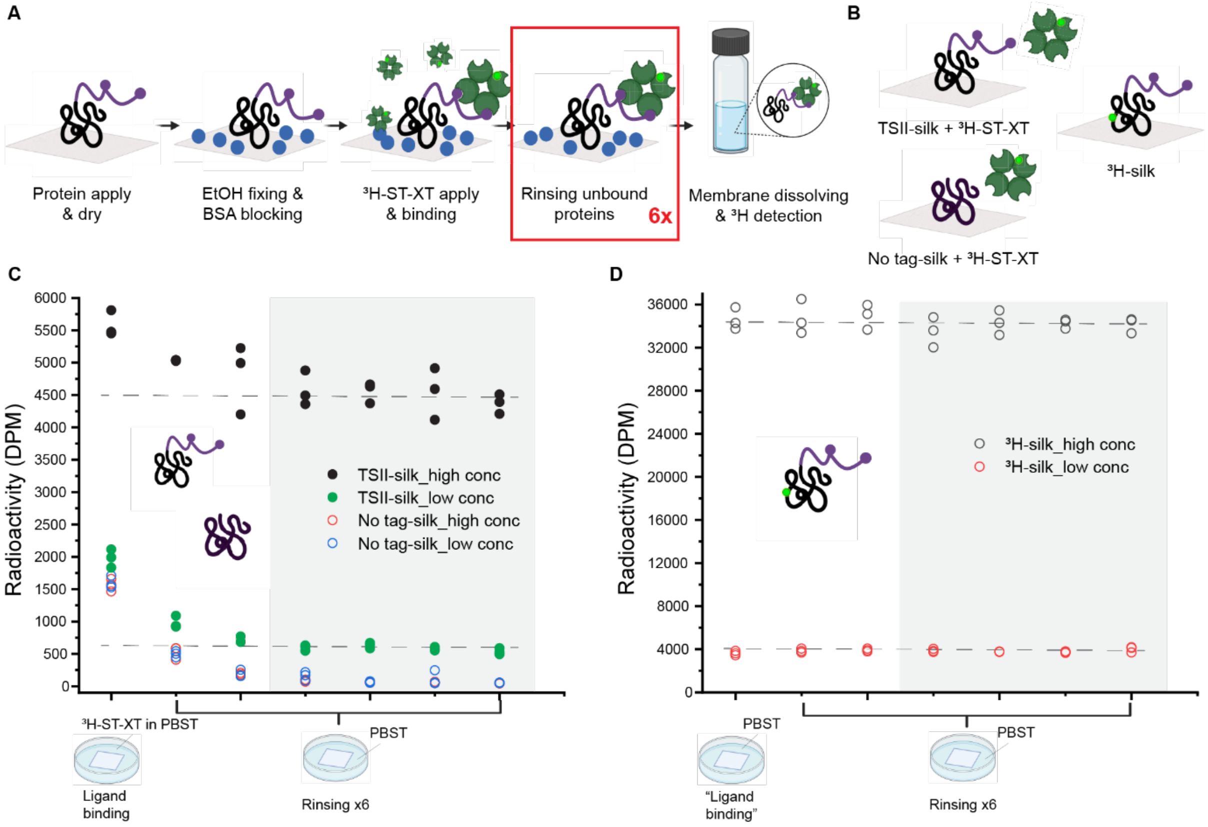

Figure S3. Optimization of rinsing of non-specifically bound probe. A) Schematic of experimental steps; the full assay was conducted with additional rinsing steps up to 6 times. B) Proteins used; TSII-silk compared to negative control (no tag-silk) with labelled Strep-TactinXT and as comparison ^{3}H-silk. C) Rinsing non-specifically bound Strep-TactinXT. After 3 rounds of rinsing the activity is stable with a similar trend at different concentrations of proteins. D) The stability of ^{3}H-silk over the rinses shows that the decrease of activity in panel C results is from loss of Strep-TactinXT and not loss of silk proteins.

Location of non-specific binding on sample membranes

To understand better the nature of non-specific binding in our assay, its location was studied. A normal assay was conducted for Twin-Strep-silk and negative control (no tag) silk with ^{3}H-Strep-TactinXT and samples were measured from both directly after probe binding (no rinsing) and after the whole assay (3 rinsing rounds). The location was studied by cutting one sample into 5x5 squares, resulting in 25 samples per original sample membrane (figure S4A). These small membrane pieces were separately measured.

In Twin-Strep-silk samples, most of the activity is in the middle of the membrane, where the silk was applied originally (figure S4B, column 1, red). Directly after Strep-TactinXT binding there is notable activity also on the edges of the membrane (row 1), but after rinsing, the activity is decreased to very low values (column 1, row 2). The activity in the middle of the membrane is slightly decreased during rinsing, but values are still high, indicating that the specifically bound Strep-TactinXT are staying bound during rinsing and the non-specific binding occurs mostly on the edges of the membrane, where no silk is present, but the rinsing removes it efficiently. This is confirmed by the negative control samples (column 2, blue). Directly after Strep-TactinXT binding (column 2, row 1) the activity overall is very low, but most of the activity is on the edges where no silk is present but mostly BSA-blocked membrane. The silk structure itself does not seem to bind Strep-TactinXT non-specifically. After rinsing (column 2, row 2), the activity is decreased in all parts of the membrane close to zero confirming that the rinsing is efficient. The activity is evenly distributed on the whole membrane declaring no difference in the remaining non-specific binding between silk and BSA blocked membrane.

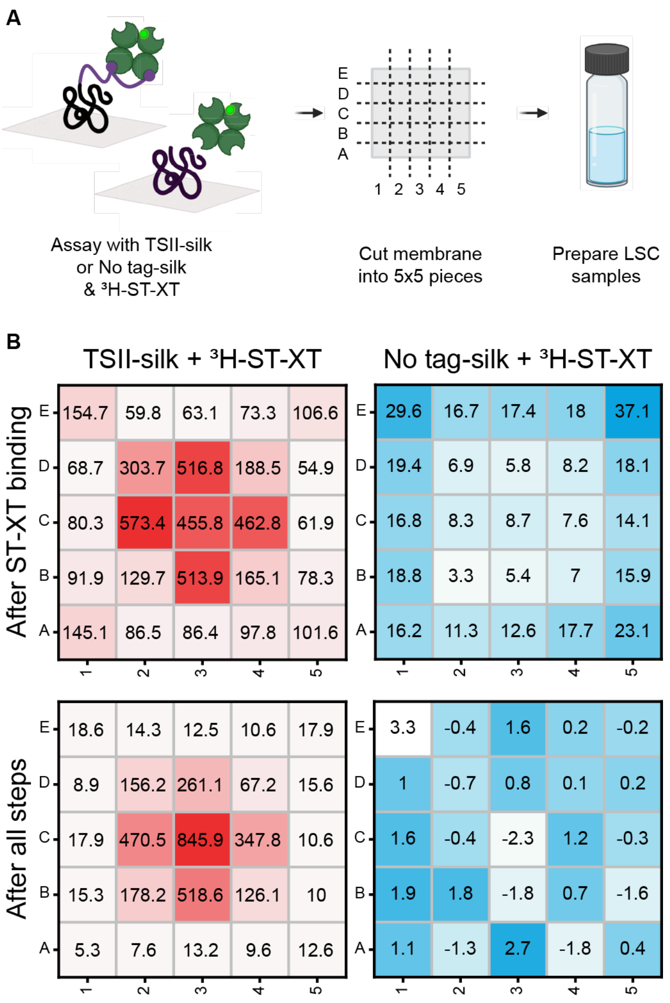

Figure S4. Location of non-specific binding on sample membrane. A) Schematic of the experimental setup. B) Heat maps of activity comparing TSII-silk and negative control silk, and the location of non-specific binding directly after Strep-TactinXT binding and after all rinsing steps.

Calculations of validation parameters

To validate the developed method limit of detection (LOD), limit of quantification (LOQ), linearity, linear region, accuracy, and precision were determined. The limit of detection and limit of quantification were determined from the background signal (negative control) of the standard by signal-to-noise ratio method. The limit of detection was determined with Equation 1

$$LOD = BG + 3 * \sigma \quad (1)$$

And the limit of quantification was determined in similar way by Equation 2

$$LOQ = BG + 10 * \sigma \quad (2)$$

Where in both BG is the average background signal value and σ is the standard deviation of the background signal.

Linearity and linear range of detection were determined with the Analysis of Variance lack-of-fit (LOF) test[7]. The sum of least squares method was used to determine the linear regression curve (Equation 3) for the standard sample series. Equation 3 is presented below

$$\hat{y}_i = a * x_i + b \quad (3)$$

Where $\hat{y}_i$ is the estimated experimental response and x_i is the analytical concentration both at a concentration level of i. The coefficient a represents the slope (sensitivity of analysis) and b intercept. In preparation for the LOF test, error sum squares were calculated from the standard sample series. The residual error sum of squares (SSr, Eq 4), pure experimental error sum of squares (SSe, Eq 5) and lack-of-fit error sum of squares (SSlof, Eq 6) were calculated according to the following equations:

$$SS_r = \sum_{i=1}^{I} \sum_{j=1}^{J_i} (y_{ij} - \hat{y}_i)^2 \quad (4)$$

$$SS_e = \sum_{i=1}^{I} \sum_{j=1}^{J_i} (y_{ij} - \bar{y}_i)^2 \quad (5)$$

$$SS_{lof} = SS_r - SS_e = \sum_{i=1}^{I} (\bar{y}_i - \hat{y}_i)^2 \quad (6)$$

Where y_{ij} represents the experimental response, $\hat{y}_i$ represents the estimated response obtained from the linear regression curve (Eq 3), and $\bar{y}_i$ is the average response at every concentration level i. I represents the number of concentration levels and J the number of replicates in each concentration. The degrees of freedom (DF) associated with equations 4-6 are calculated with the following equations respectively:

$$DF_r = (IJ - 2) \quad (7)$$

$$DF_e = (IJ - I) \quad (8)$$

$$DF_{lof} = (I - 2) \quad (9)$$

Next the purely experimental variance (σ_e^2) and lack-of-fit variance (σ_{lof}^2) were calculated with the following equations respectively:

$$\sigma_e^2 = \frac{SS_e}{DF_e} \quad (10)$$

$$\sigma_{lof}^2 = \frac{SS_{lof}}{DF_{lof}} \quad (11)$$

With these variance terms the experimental Fisher variance ratio or F-test value was calculated with the following equation:

$$F_{(I-2)/(IJ-I)} = \frac{\sigma_{lof}^2}{\sigma_e^2} \quad (12)$$

This experimentally calculated $F_{(I-2)/(IJ-I)}$ value was then compared against the critical value of F from statistical tables. The 95 % confidence level with degrees of freedom $I - 2$ for the numerator and $IJ - I$ for the denominator was used for the critical value. The null hypothesis (H_0) of the test is that there is no lack-of-fit (regression is linear) whereas the alternative hypothesis (H_A) states that lack-of-fit is present, and some non-linear regression should be applied. Therefore, when the calculated F-value is smaller than the tabulated F-value, the null hypothesis is true, and a linear equation best describes the data[7,8].

Finally, the accuracy and precision were determined. Accuracy was determined from the relative error of estimated concentration when compared to a known value of reference determined independently by amino acid analysis and verified by UV-visible spectroscopy before each experiment.[7] The estimated concentration value ($\hat{x}_i$, Eq 13) was determined from the experiment specific standard linear curve equation (Eq 3) as presented below:

$$\hat{x}_i = \frac{(y_{ij} - b_k)}{a_k} \quad (13)$$

Where a_k present the experiment specific standard curve slope value and b_k the experiment specific intercept value.
The relative error (RE) was calculated with Equation 14

$$RE = \left|\frac{\hat{x}_i}{x_i}\right| * 100 - 100 \quad (14)$$

From where the final accuracy value (%) was obtained with Equation 15 using the average of all individually defined relative error values:

$$Accuracy = 100 - RE_{ave} \quad (15)$$

Precision was determined from the relative standard deviation ($\sigma_{\%}$) of replicate samples presented in Equation 16

$$\sigma_{\%} = \frac{\sigma}{\bar{y}_i} * 100 \quad (16)$$

From where the final precision value (%) was obtained with Equation 17 using the average of all individually defined relative standard deviation values:

$$Precision = 100 - \sigma_{\%,ave} \quad (17)$$

The final limit of detection, limit of quantification, accuracy, and precision values were presented with standard error (SE) values, which were calculated with Equation 18

$$SE = \frac{\sigma}{\sqrt{n}} \quad (18)$$

Where σ is the standard deviation of the final LOD, LOQ, accuracy, or precision, and n is the number of samples in the calculation. The data for our validation calculations is available at Zenodo.org: DOI: 10.5281/zenodo.12579698.

References

(1) Huemmerich, D.; Helsen, C. W.; Quedzuweit, S.; Oschmann, J.; Rudolph, R.; Scheibel, T. Primary Structure Elements of Spider Dragline Silks and Their Contribution to Protein Solubility. *Biochemistry* **2004**, *43* (42), 13604–13612. https://doi.org/10.1021/bi048983q.

(2) Tormo, J.; Lamed, R.; Chirino, A. J.; Morag, E.; Bayer, E. A.; Shoham, Y.; Steitz, T. A. Crystal Structure of a Bacterial Family-III Cellulose-Binding Domain: A General Mechanism for Attachment to Cellulose. *EMBO J.* **1996**, *15* (21), 5739–5751. https://doi.org/10.1002/j.1460-2075.1996.tb00960.x.

(3) Mohammadi, P.; Aranko, A. S.; Lemetti, L.; Cenev, Z.; Zhou, Q.; Virtanen, S.; Landowski, C. P.; Penttilä, M.; Fischer, W. J.; Wagermaier, W.; Linder, M. B. Phase Transitions as Intermediate Steps in the Formation of Molecularly Engineered Protein Fibers. *Commun. Biol.* **2018**, *1* (1), 86. https://doi.org/10.1038/s42003-018-0090-y.

(4) Malkamäki, M. Engineering Flax-Protein Composites. Master's thesis, Aalto University, Espoo, 2020.

(5) Beamson, G.; Briggs, D. High Resolution XPS of Organic Polymers - The Scienta ESCA300 Database Wiley Interscience; 1992.

(6) Justin Gorham. NIST X-Ray Photoelectron Spectroscopy Database - SRD 20, 2012. https://doi.org/10.18434/T4T88K.

(7) Araujo, P. Key Aspects of Analytical Method Validation and Linearity Evaluation. *J. Chromatogr. B* **2009**, *877* (23), 2224–2234. https://doi.org/10.1016/j.jchromb.2008.09.030.
(8) Raposo, F. Evaluation of Analytical Calibration Based on Least-Squares Linear Regression for Instrumental Techniques: A Tutorial Review. *TrAC Trends Anal. Chem.* **2016**, *77*, 167–185. https://doi.org/10.1016/j.trac.2015.12.006.
